# Inhibition of Human Coronaviruses by Combinations of Host-Targeted and Direct-Acting Antivirals

**DOI:** 10.1128/aac.01703-22

**Published:** 2023-03-28

**Authors:** Patricia de León, Rodrigo Cañas-Arranz, María José Bustos, Margarita Sáiz, Francisco Sobrino

**Affiliations:** a Centro de Biología Molecular “Severo Ochoa” (CSIC-UAM), Universidad Autónoma de Madrid, Madrid, Spain

**Keywords:** coronavirus, SARS-CoV-2, host-targeted antivirals, direct-acting antivirals, lauryl gallate, valproic acid, remdesivir, synergy

## Abstract

Antiviral compounds targeting cellular metabolism are part of the therapeutic arsenal to control the spread of virus infection, either as sole treatment or in combination with direct-acting antivirals (DAA) or vaccines. Here, we describe the effect of two of them, lauryl gallate (LG) and valproic acid (VPA) both exhibiting a wide antiviral spectrum, against infection by coronaviruses such as HCoV-229E, HCoV-OC43, and SARS-CoV-2. A consistent 2 to 4-log-decrease in virus yields was observed in the presence of each antiviral, with an average IC_50_ value of 1.6 μM for LG and 7.2 mM for VPA. Similar levels of inhibition were observed when adding the drug 1 h before adsorption, at the time of infection or 2 h after infection, supporting a postvirus entry mechanism of action. The specificity of the antiviral effect of LG against SARS-CoV-2, relative to other related compounds such as gallic acid (G) and epicatechin gallate (ECG), predicted to be better inhibitors according to *in silico* studies, was also demonstrated. The combined addition of LG, VPA, and remdesivir (RDV), a DAA with a proven effect against human coronaviruses, resulted in a robust synergistic effect between LG and VPA, and to a lesser extent between the other drug combinations. These findings reinforce the interest of these wide antiviral spectrum host-targeted compounds as a first line of defense against viral diseases or as a vaccine complement to minimize the gap in antibody-mediated protection evoked by vaccines, either in the case of SARS-CoV-2 or for other possible emerging viruses.

## INTRODUCTION

Compounds targeting cellular functions required for viruses to complete their multiplication cycle, instead of virus-specific targets, are an interesting approach for preventing and controlling virus infection (1). An efficient host-targeted antiviral (AV) would inhibit viral entry and/or replication at nontoxic concentrations for the cell and, ideally, it would help to control a wide variety of viral diseases and virus strains and could be administered alone, in combination with other AVs, or as a complement to vaccination (1, 2). Selection of virus-escape mutants, which can overcome the activity of inhibitors posing a crucial Achilles’ heel of AV compounds (3, 4) is expected to be less prone for host-targeted AVs (5, 6). Simultaneous administration of two inhibitors directed to independent targets is also reported to increase the barrier to resistance for selection of virus-escape mutants (7–9). Thus, delivery of combinations of broad-spectrum AVs directed to different therapeutic targets is emerging as a valuable strategy for the control of virus infection (10, 11). We have described the ability of two host-targeted compounds, lauryl gallate (LG) and valproic acid (VPA), to inhibit virus multiplication. LG is an ester derivative of gallic acid (G), a natural plant fatty phenolic compound. Alkyl (propyl, octyl, and lauryl) esters of G, known as food additives (E-310, E-311, and E-312, respectively), have been widely used as antioxidants, as well as part of pharmaceutical formulations (12). Esters of G have also been reported to inhibit the proliferation of tumoral cell lines (13) by triggering the apoptosis of the cell (14) and the concomitant inhibition of protein tyrosine kinases (15) and have been described as AVs (16–18). We have previously reported that LG can inhibit the replication in cultured cells of African swine fever virus (ASFV), foot-and-mouth disease virus (FMDV), herpesvirus simplex (HSV-I), influenza virus, Sindbis virus (SINV), vaccinia virus (VACV), vesicular stomatitis virus (VSV), swine vesicular disease virus (SVDV), and transmissible gastroenteritis virus (TGEV), also reducing mortality and FMDV load *in vivo* in a mouse model (19, 20). The antiviral effect of LG might be mediated by its interaction with phospholipids in biological membranes (21), with surfactant effects destabilizing the cellular and viral envelopes. Likewise, VPA, a branched short-chain fatty acid with a known therapeutic role against neurological diseases such as epilepsy, migraines, or bipolar disorders (22), inhibits the multiplication in cultured cells of a wide variety of enveloped viruses, including VSV, West Nile virus (WNV), human herpesvirus type I (HHV-1), ASFV, Semliki forest virus (SFV), SINV, Usutu virus (USUV), lymphocytic choriomeningitis virus (LCMV), and HSV-I (23–25). A retrospective study has also revealed a reduced risk of clinical infection by herpesviruses in patients exposed to VPA (26). VPA has been shown to alter a variety of signaling pathways, including an increase in GABA neurotransmission and inhibition of glycogen synthase kinase beta (GSK3β), or attenuation of phospholipid signaling (22). In addition, the VPA class I HDAC (histone deacetylase) inhibitory activity has been associated with an anti-inflammatory effect in mice (27) and therapeutic roles of VPA have been proposed in cancer, Alzheimer´s disease, and human immunodeficiency virus (HIV) treatment (28).

Since its burst in early 2020, the coronavirus disease pandemic (COVID-19), which is caused by severe acute respiratory syndrome coronavirus 2 (SARS-CoV-2) (29), has killed over six million people worldwide and produced unknown long-term clinical signs among people with different age and health conditions (https://covid19.who.int/). This unprecedented global shock has triggered the extensive search for AV compounds that efficiently inhibit SARS-CoV-2 infection and mitigate COVID-19 clinical signs (30). Yet there are still limited therapeutic options for treating this disease, which underline the need for the identification of new AV candidates, as well as for optimizing delivery strategies. Thus, combined administration of drugs with independent mechanisms of action could result in synergy against SARS-CoV-2, improving antiviral efficacy (10). VPA and LG are generic widely used drugs with low cost and ready for use in humans. In the context of the COVID-19 pandemic, VPA has been proposed as a candidate for drug repurposing based on the analysis of the interaction landscape of SARS-CoV-2 proteins and druggable human proteins (31). In endothelial cells, VPA can decrease pulmonary lipopolysaccharide (LPS)-induced lung inflammation and inhibit the expression of angiotensin-converting enzyme (ACE-2) receptors that are the entry door for SARS-CoV-2 into the cells. In addition, VPA-treated patients seem to develop less serious COVID-19 than control patients, according to diverse clinical endpoints and laboratory markers (32). Likewise, LG has been identified, among other compounds, as a potential anti-SARS-CoV-2 drug, based on docking experiments (binding affinity) (33). In this scenario, we have tested the effect of LG, VPA, and their combination on human coronaviruses (HCoVs): the *Alphacoronavirus* HCoV-229E, the *Betacoronavirus* HCoV-OC43, both associated with common cold, and the *Betacoronavirus* SARS-CoV-2, responsible for the ongoing COVID-19 pandemic (34). The effect of the combined administration of LG, VPA and the direct-acting antiviral (DAA) remdesivir (RDV), an adenosine analogue that inhibits SARS-CoV-2 multiplication (35) and exerts a broad antiviral activity, including HCoVs, has also been evaluated.

Our data indicate that both host-targeted drugs, LG and VPA, exhibit a wide and consistent antiviral spectrum against the multiplication of coronaviruses, such as HCoV-229E, HCoV-OC43, and SARS-CoV-2, in all coronavirus-susceptible cell lines tested (average IC_50_ values of 1.6 μM for LG and 7.2 mM for VPA). Interestingly, high synergistic effects at nontoxic concentrations of the combined drugs, have been observed against the replication of the three HCoVs analyzed, mainly for LG in combination with VPA or RDV and for the three compounds together. In summary, these results expand the repertoire of therapeutic compounds potentially useful for coronavirus control, including that of SARS-CoV-2.

## RESULTS

### Direct virotoxicity of cell-targeted AVs on HCoVs.

Direct virucidal effect of LG and VPA in coronavirus particles was assayed by incubation of virus samples (10^5^ PFU) for 1 h at room temperature with increasing concentrations of the drugs (0 to 300 μM for LG, or 0 to 300 mM for VPA). No decrease in virus titers were observed in HCoV-229E-GFP, HCoV-OC43, or SARS-CoV-2 samples when incubated with the compounds in the range of concentrations analyzed (Fig. S1).

### Inhibition of HCoVs by different AVs.

We analyzed the effect of LG and VPA in cells infected with different HCoVs (HCoV-229E-GFP, HCoV-OC43, or SARS-CoV-2). The antiviral effect of RDV alone was also examined in this experiment as reference for the synergy experiments described below.

We first assayed the cytotoxicity of LG, VPA, or RDV in different cell lines. Cultures of Huh-7 (male hepatoma tissue) for HCoV-229E-GFP and HCoV-OC43; Calu3 2B4 cells (human broncho-epithelial) for SARS-CoV-2 Wuhan-Hu-1 strain; and Vero E6 for SARS-CoV-2 omicron strain were incubated for 24 h (for HCoV-229E-GFP and SARS-CoV-2) or 48 h (for HCoV-OC43) in the presence of increasing concentrations of the AVs, and CC_50_ values were determined (Table 1).

**TABLE 1:** Formula and cytotoxicity of AVs in different cell lines

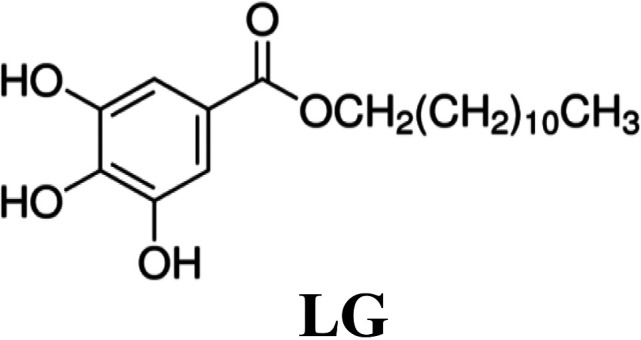		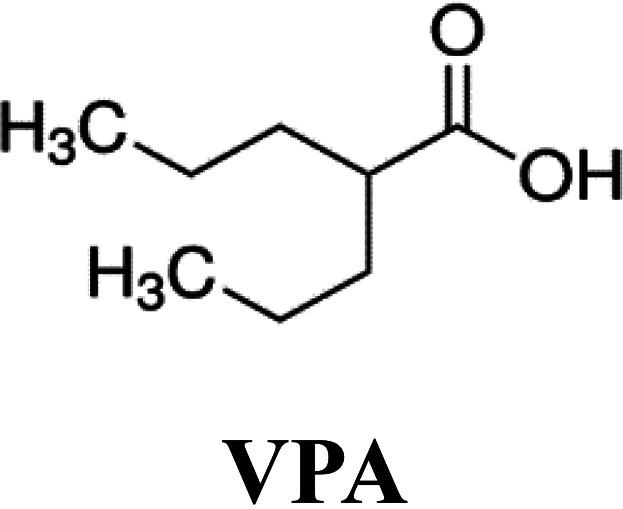	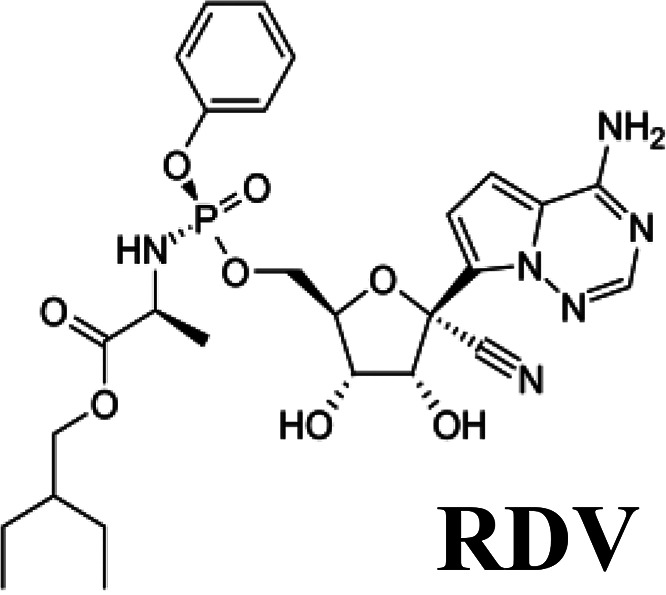
	AV^*a*^		
Cell line	LG	VPA	RDV
Huh-7	83.1 μM^*b*^	70 mM^*b*^	25 μM^*b*^
	55 μM^*c*^	66 mM^*c*^	20 μM^*c*^
HCT-8	55 μM^*b*^	95.7 mM^*b*^	43 μM^*b*^
	50.4 μM^*c*^	76.2 mM^*c*^	38 μM^*c*^
Vero E6	90.9 μM^*b*^	111 mM^*b*^	85 μM^*b*^
Calu3 2B4	123.7 μM^*b*^	124 mM^*b*^	109 μM^*b*^

a Data expressed as CC_50_, concentration of antiviral yielding a 50% of cell viability in MTT assay.

b 24 h cell incubation with the corresponding AV.

c 48 h cell incubation with the corresponding AV.

**FIG 1:**
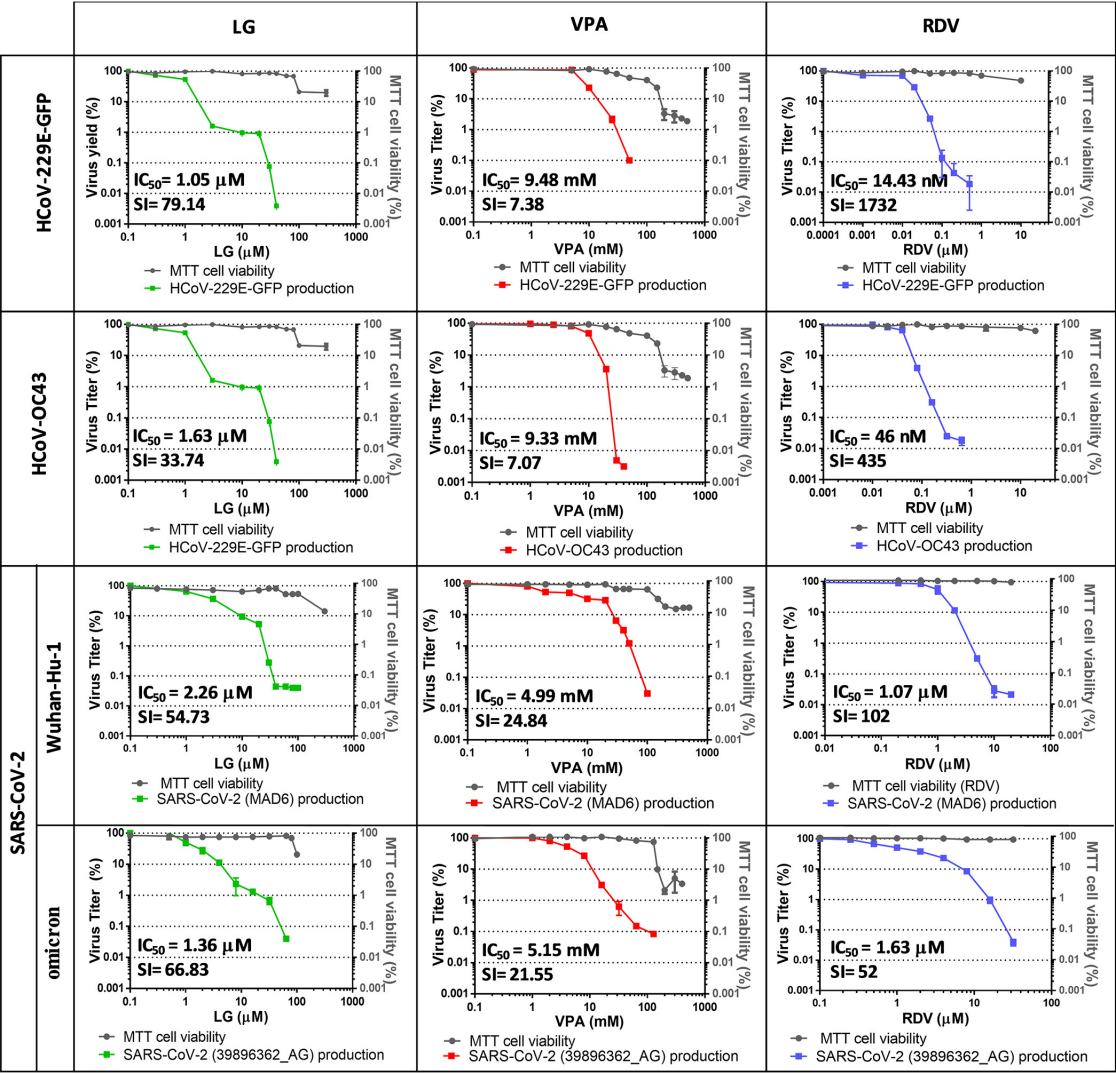
Inhibitory effect of LG, VPA, or RDV against HCoVs. Three replicates of antiviral assays in the presence of LG, VPA, or RDV were performed on triplicate cultures of Huh-7 cells infected with HCoV-229E-GFP or HCoV-OC43, and on Calu-3 2B4 infected with SARS-CoV-2 isolate MAD-6 (Wuhan-Hu-1 strain) or Vero E6 with SARS-CoV-2 isolate 39896362_AG (omicron strain). Incubation with the AV was carried out from 1 h before adsorption and maintained throughout the infection. The left *y* axis represents viral titers at 24 hpi (for HCoV-229E-GFP and SARS-CoV-2) or 48 hpi (for HCoV-OC43), determined by plaque assays on Huh-7 cells for HCoV-229E-GFP, HCT-8 cells for HCoV-OC43, or Vero E6 for SARS-CoV-2. The right *y* axis represents cell viability determined by MTT assay of each corresponding cell line. IC_50_ and SI values are displayed. HCoV-229E-GFP titers (PFU/mL): 3 ± 0.5 × 10^6^ PFU/mL in the absence of the drugs, 1.4 ± 0.2 × 10^2^ at LG 40 μM, 2.5 ± 0.3 × 10^2^ at VPA 40 mM or 6 ± 0.1 × 10^2^ at RDV 500 nM. HCoV-OC43 titers (PFU/mL): 3.1 ± 0.9 × 10^6^ in the absence of the drugs, 5 ± 0.01 × 10^1^ at LG 40 μM, 1.2 ± 0.1 × 10^2^ at VPA 40 mM or 6 ± 0.1 × 10^2^ at RDV 500 nM. SARS-CoV-2 isolate MAD6 titers (PFU/mL): 3.4 ± 1 × 10^5^ in the absence of the drugs, 5 ± 0.01 × 10^1^ at LG 100 μM, 5 ± 0.01 × 10^1^ at VPA 100 mM or 6 ± 0.3 × 10^2^ at RDV 20 μM. SARS-CoV-2 isolate 39896362_AG titers (PFU/mL): 1 ± 0.1 × 10^5^ in the absence of the drugs, 5 ± 0.01 × 10^1^ at LG 64 μM, 1 ± 0.1 × 10^2^ at VPA 128 mM or 5 ± 0.01 × 10^1^ at RDV 32 μM.

When assays were conducted adding the AV 1 h before adsorption and maintaining AV incubation throughout the infection, a consistent 2 to 4 log decrease was observed in the production of all tested HCoVs in the presence of nontoxic concentrations of LG, with an average IC_50_ value of 1.6 μM (IC_50_ = 1.05 ± 0.3 μM for HCoV-229E-GFP, 1.63 ± 0.6 μM for HCoV-OC43, 2.26 ± 0.5 μM for SARS-CoV-2 Wuhan-Hu-1 strain, and 1.36 ± 0.2 μM for SARS-CoV-2 omicron strain) (Fig. 1). Likewise, treatment with increasing concentrations of VPA that did not affect cell viability, lead to a 2 to 4-log decrease in coronaviruses multiplication, with IC_50_ values that resulted lower for SARS-CoV-2 (4.99 ± 0.2 mM for the Wuhan-Hu-1 strain and 5.15 ± 0.3 for the omicron strain) than for HCoV-229E-GFP (9.48 ± 1.1 mM) or HCoV-OC43 (9.33 ± 2.1 mM). Treatment with the DAA compound RDV reduced virus yields from all coronaviruses analyzed, with IC_50_ values (from 14.43 ± 4.1 nM for HCoV-229E-GFP to 1.63 ± 0.2 μM for SARS-CoV-2 omicron strain) similar to those previously reported (36, 37). The selectivity index (SI) allows estimation of the window between cytotoxicity and AV activity of a given compound; the higher the ratio (values ≥ 10), the safer and more effective that would be for treatment of a viral infection (38, 39). LG lead to high SI values, above 30, for all tested HCoVs (from 33.74 for HCoV-OC43 to 79.14 for HCoV-229E-GFP). In the case of VPA, SI values slightly above 7 for HCoV-229E-GFP and HCoV-OC43 indicated moderate antiviral effectiveness, while a strong inhibition, SI above 20, was observed for Wuhan-Hu-1 and omicron SARS-CoV-2 strains (Fig. 1). Thus, a solid inhibitory potential was found for LG and VPA, regardless of the HCoV or the SARS-CoV-2 strain analyzed.

On the other hand, AV assays confirmed that LG and VPA inhibited the multiplication of the swine coronavirus TGEV in a parallel manner (Fig. S2).

### Effect of time of addition of host-targeted AVs.

**FIG 2:**
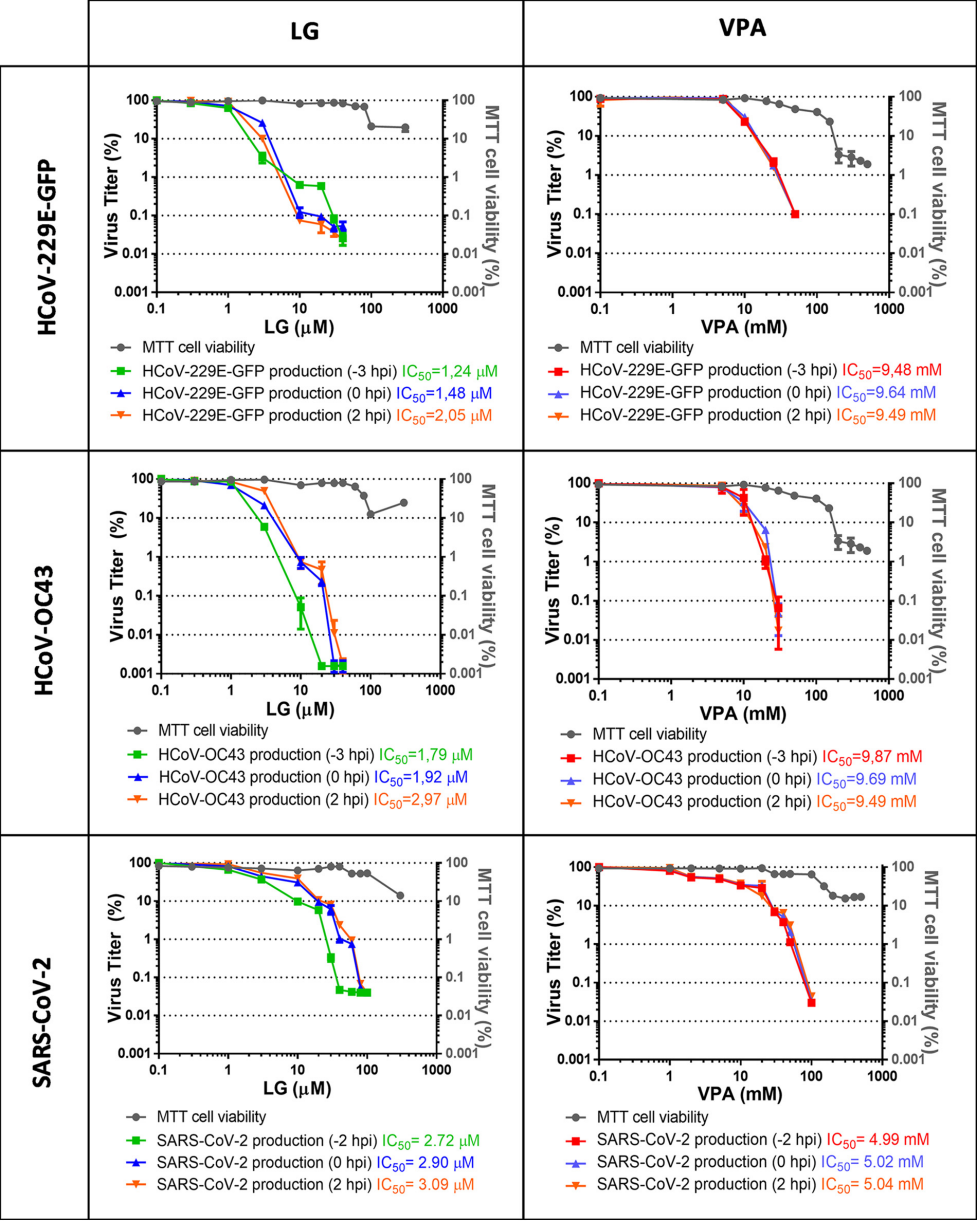
Effect of the time of addition of the AV during infection. Triplicate cell cultures were infected with the different coronaviruses and the AV was added 1 h before adsorption (−3 hpi for HCoV-229E-GFP and HCoV-OC43 or −2 hpi for SARS-CoV-2 isolate MAD6 [Wuhan-Hu-1 strain]), at the time of infection (0 hpi) or 2 h after infection (2 hpi). Incubation with the AVs was maintained throughout the infection. Left *y* axis represents viral titers at 24 hpi (for HCoV-229E-GFP and SARS-CoV-2) or 48 hpi (for HCoV-OC43), determined by plaque assays on triplicate cultures of Huh-7 cells for HCoV-229E-GFP, HCT-8 cells for HCoV-OC43, or Vero E6 for SARS-CoV-2. The right *y* axis represents cell viability determined by MTT assay of each corresponding cell line. IC_50_ values are displayed. Three replicates were made for each antiviral trial. HCoV-229E-GFP average titers (PFU/mL): 4 ± 0.6 × 10^6^ in the absence of the drugs, 6 ± 2 × 10^2^ at 40 μM LG or 40 mM VPA. HCoV-OC43 average titers (PFU/mL): 3.5 ± 0.5 × 10^6^ in the absence of the drugs, 5 ± 0.01 × 10^1^ at 40 μM LG or 40 mM VPA. SARS-CoV-2 average titers (PFU/mL): 3.4 ± 0.2 × 10^5^ in the absence of the drugs, 5 ± 0.01 × 10^1^ at 100 μM LG or 100 mM VPA.

The effect of the time of addition of the AVs on viral titers, important information to be considered for antiviral drug delivery, was assayed with HCoV-229E-GFP, HCoV-OC43, and SARS-CoV-2 (Wuhan-Hu-1 strain). In the case of LG, no major changes in the inhibition levels were observed when it was added 1 h before adsorption, at the time of infection, or 2 h after infection (Fig. 2). Nevertheless, IC_50_ values slightly increased as the drug was added later in the infection, being this difference was higher for HCoV-OC43 (from 1.79 μM [−3 hpi] to 2.97 μM [2 hpi]) and rather low for SARS-CoV-2 (from 2.72 μM [-2 hpi] to 3.09 μM [2 hpi]). This result suggests that LG might affect both entry and postentry steps of infection cycle. For VPA, nearly identical IC_50_ values were determined when adding the drug before, at the time or after infection for the three coronaviruses tested (Fig. 2). This result suggests that HCoV entry is not affected by VPA.

### Specificity of the inhibition exerted by LG on HCoV infections.

**FIG 3:**
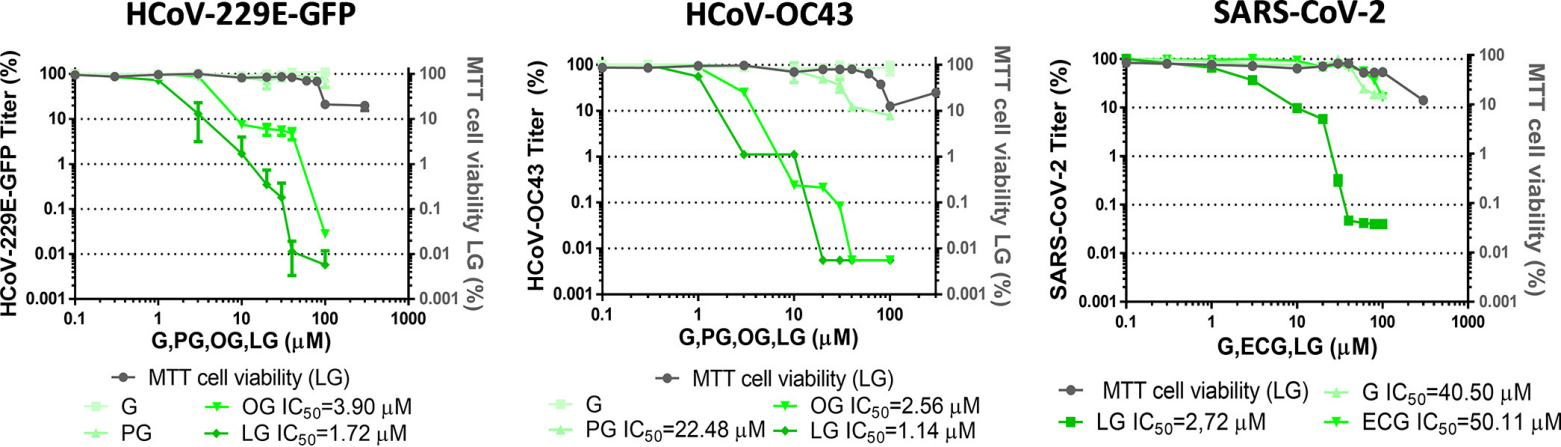
Specificity of the inhibition exerted by LG on coronavirus infection. The inhibitory effect of LG among other related compounds was assayed on triplicate cultures of Huh-7 cells infected with HCoV-229E-GFP or HCoV-OC43, and on Calu-3 2B4 infected with SARS-CoV-2 isolate MAD6 (Wuhan-Hu-1 strain). Incubation with the different compounds was carried out from 1 h before adsorption and maintained throughout the infection. The left *y* axis represents viral titers at 24 hpi (for HCoV-229E-GFP and SARS-CoV-2) or 48 hpi (for HCoV-OC43), determined by plaque assays on triplicate cultures of Huh-7 cells for HCoV-229E-GFP, HCT-8 cells for HCoV-OC43, or Vero E6 for SARS-CoV-2. The right *y* axis represents cell viability determined by MTT assay of each corresponding cell line. IC_50_ values are shown in cases where it was possible to calculate them. G: gallic acid P: propyl gallate; OG: octyl gallate; ECG: epicatechin gallate. Three replicates were made for each antiviral trial. HCoV-229E-GFP titers (PFU/mL): 4 ± 0.9 × 10^6^ in the absence of the drugs, 3.1 ± 0.2 × 10^6^ at 100 μM G, 2.2 ± 0.3 × 10^6^ at 100 μM PG, 1.4 ± 0.5 × 10^3^ at 100 μM OG or 5 ± 0.2 × 10^2^ at 100 μM LG. HCoV-OC43 titers (PFU/mL): 1.8 ± 0.9 × 10^6^ in the absence of the drugs, 1.2 ± 0.2 × 10^6^ at 100 μM G, 1.2 ± 0.3 × 10^6^ at 100 μM PG, 5 ± 0.01 × 10^1^ at 100 μM OG or 5 ± 0.01 × 10^1^ at 100 μM LG. SARS-CoV-2 titers (PFU/mL): 3.±0.1 × 10^5^ in the absence of the drugs, 7.5 ± 0.2 × 10^4^ at 100 μM G, 6 ± 0.3 × 10^4^ at 100 μM ECG or 1 ± 0.1 × 10^2^ at 100 μM LG.

The specificity of the inhibition exerted by LG among the other related compounds, G and its esters, PG and OG, reported to have antiviral effects (17, 18) was first analyzed in HCoV-229E-GFP- and HCoV-OC43-infected Huh-7 cells. LG was the compound that most efficiently inhibited both infections, resulting in a 2 to 4-log-decrease in virus yields, in a range of concentrations (10 to 30 μM) that still maintained cell viability (IC_50_ = 1.72 μM for HCoV-229E-GFP; IC_50_ = 1.14 μM for HCoV-OC43) (Fig. 3). In the case of HCoV-229E-GFP, OG also reduced virus multiplication but to a lesser extent, a 1 to 2-log decrease at nontoxic concentrations, with a resulting IC_50_ value higher than that of LG (OG IC_50_ = 3.9 μM). A 1 to 3-log decrease in HCoV-OC43 production was also found upon OG treatment but showing a IC_50_ higher than that of LG (OG IC_50_ = 2.56 μM). Neither G nor PG inhibited viral multiplication at nontoxic concentrations; in fact, IC_50_ values could only be calculated for PG against HCoV-OC43 (PG IC_50_ = 22.48 μM), a concentration that severely affected cell viability (Fig. 3).

In addition, LG specificity in the inhibition of SARS-CoV-2 was studied among two compounds, G and ECG, derivatives with *in silico* predicted anti-SARS-CoV-2 activity higher than LG (33). When the activity of these drugs was studied in SARS-CoV-2-infected Calu3 2B4 cells, LG was found to be the most effective AV, resulting in a 2- to 4-log decrease in virus yields (IC_50_ = 2.72 μM) compared to G (IC_50_ = 40.5 μM) or ECG (IC_50_ = 50.11 μM) (Fig. 3). These results support the need for experimental testing of the virus inhibition ability of AVs in living cells as an essential aspect for AV characterization.

### Drug combination studies.

**FIG 4:**
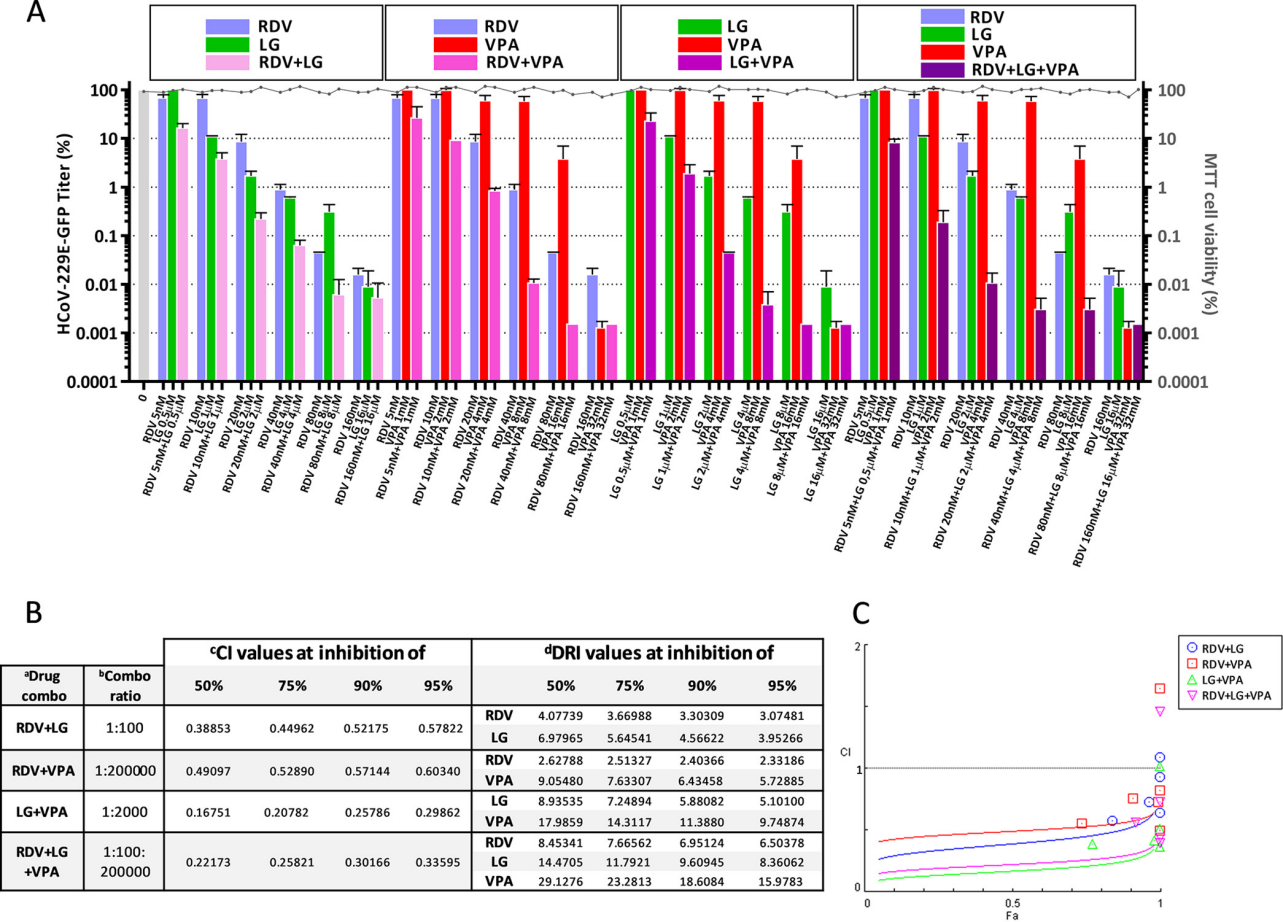
Synergistic effect of combined AVs on HCoV-229E-GFP inhibition. (A) Infectious virus progeny production determined by plaque assay (in triplicate), after infection of triplicate cultures of Huh-7 cells with HCoV-229E-GFP in the presence of one drug (LG, VPA, or RDV) or constant-ratio combinations of two or three drugs. Incubation with the AVs was carried out from 1 h before adsorption and maintained throughout the infection. (B) Synergy parameters generated by CompuSyn software (69) at 50, 75, 90, or 95% inhibition exerted by the different drug combinations (a) (drug combo), at the indicated constant-dose ratios (b) (combo ratio): synergy is determined by the combination index value (c) (CI), where CI < 1, =1, and >1 indicates synergism, additive effect, or antagonism, respectively; (d) DRI (dose-reduction index) value is folds of dose-reduction allowed for each drug, at a given degree of effect, in drug combination studies, where DRI > 1, =1, and <1 indicates favorable dose reduction, no dose-reduction, and negative dose-reduction, respectively. (C) Automated inhibition effect (Fa)-CI plots generated from the median-effect equation using CompuSyn software. Fa values (0 to 1) correspond to 0 to 100% inhibition. Three replicates were made for each antiviral trial. HCoV-229E-GFP titers (PFU/mL): 3.5 ± 0.6 × 10^6^ in the absence of the drugs, 5 ± 0.2 × 10^2^ at 16 μM LG, 5 ± 0.3 × 10^2^ at 32 mM VPA, 4 ± 0.2 × 10^2^ at 160 nM RDV, 5 ± 0.01 × 10^1^ at 16 μM LG + 32 mM VPA, 3 ± 0.2 × 10^2^ at 160 nM RDV + 16 μM LG, or 5 ± 0.01 × 10^1^ at 160 nM RDV + 32 mM VPA and 16 μM LG + 32 mM VPA + 160 nM RDV.

**FIG 5:**
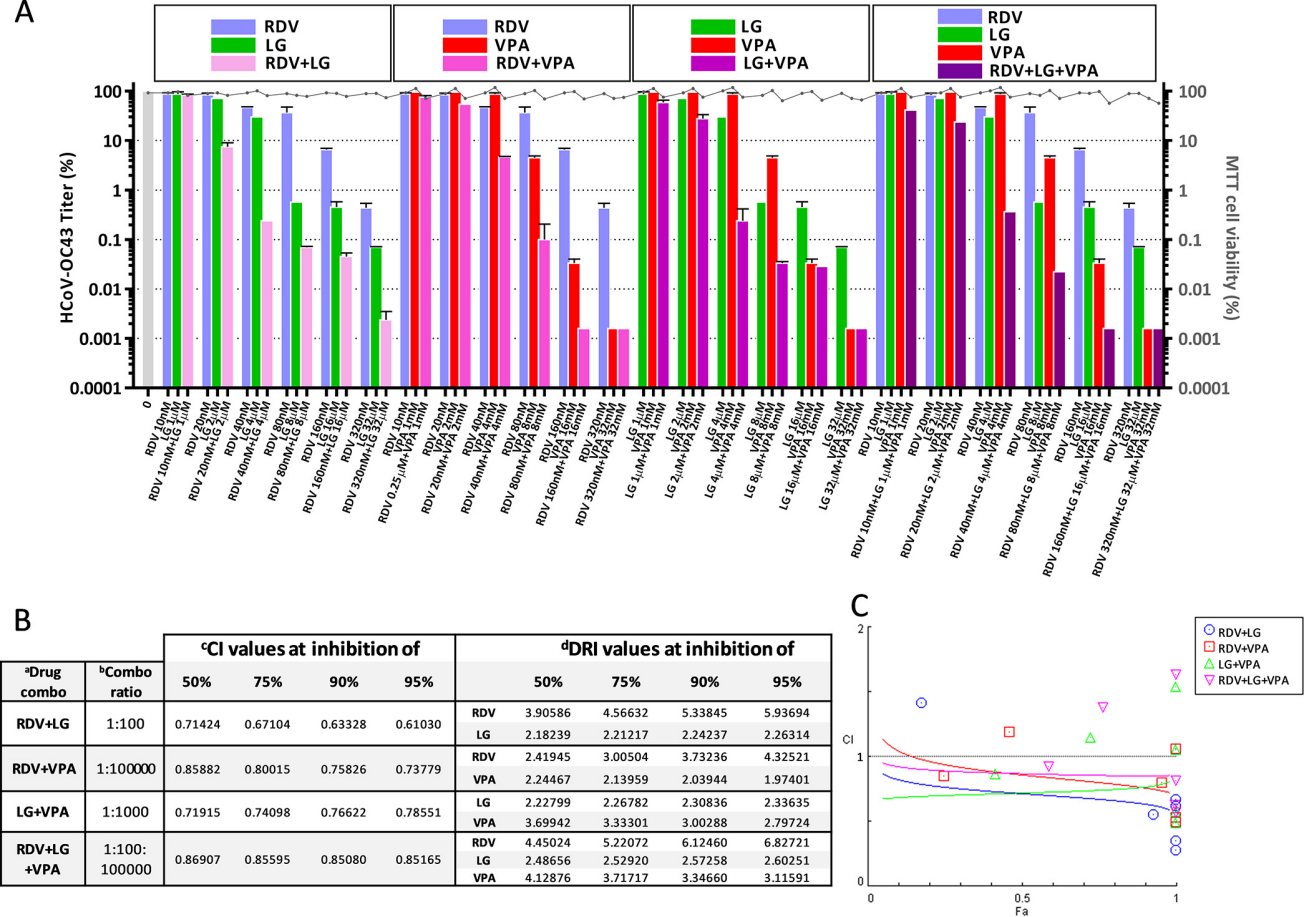
Synergistic effect of combined AVs on HCoV-OC43 inhibition. (A) Infectious virus progeny production determined by plaque assay on triplicate cultures of HCT-8 cell monolayers, after infection of triplicate cultures of Huh-7 cells with HCoV-OC43 in the presence of one drug (LG, VPA, or RDV) or constant-ratio combinations of two or three drugs. Incubation with the AVs was carried out from 1 h before adsorption and maintained throughout the infection. (B) Synergy parameters generated by CompuSyn software (69) at 50, 75, 90, or 95% inhibition exerted by the different drug combinations (a) (drug combo), at the indicated constant-dose ratios (b) (combo ratio): synergy is determined by the combination index value (c) (CI), where CI < 1, =1 and >1 indicates synergism, additive effect, or antagonism, respectively; (d) DRI (dose-reduction index) value is folds of dose-reduction allowed for each drug, at a given degree of effect, in drug combination studies, where DRI > 1, =1, and <1 indicates favorable dose reduction, no dose-reduction, and negative dose-reduction, respectively. (C) Automated inhibition effect (Fa)-CI plots generated from the median-effect equation using CompuSyn software. Fa values (0 to 1) correspond to 0 to 100% inhibition. Three replicates were made for each antiviral trial. HCoV-OC43 titers (PFU/mL): 3.2 ± 0.2 × 10^6^ PFU/mL in the absence of the drugs, 2.2 ± 0.2 × 10^3^ at 32 μM LG, 5 ± 0.01 × 10^1^ at 32 mM VPA, 1.6 ± 0.2 × 10^4^ at 320 nM RDV, or 5 ± 0.01 × 10^1^ at 32 μM LG + 32 mM VPA, 320 nM RDV + 32 μM LG, 320 nM RDV + 32 mM VPA and 32 μM LG + 32 mM VPA + 320 nM RDV.

**FIG 6:**
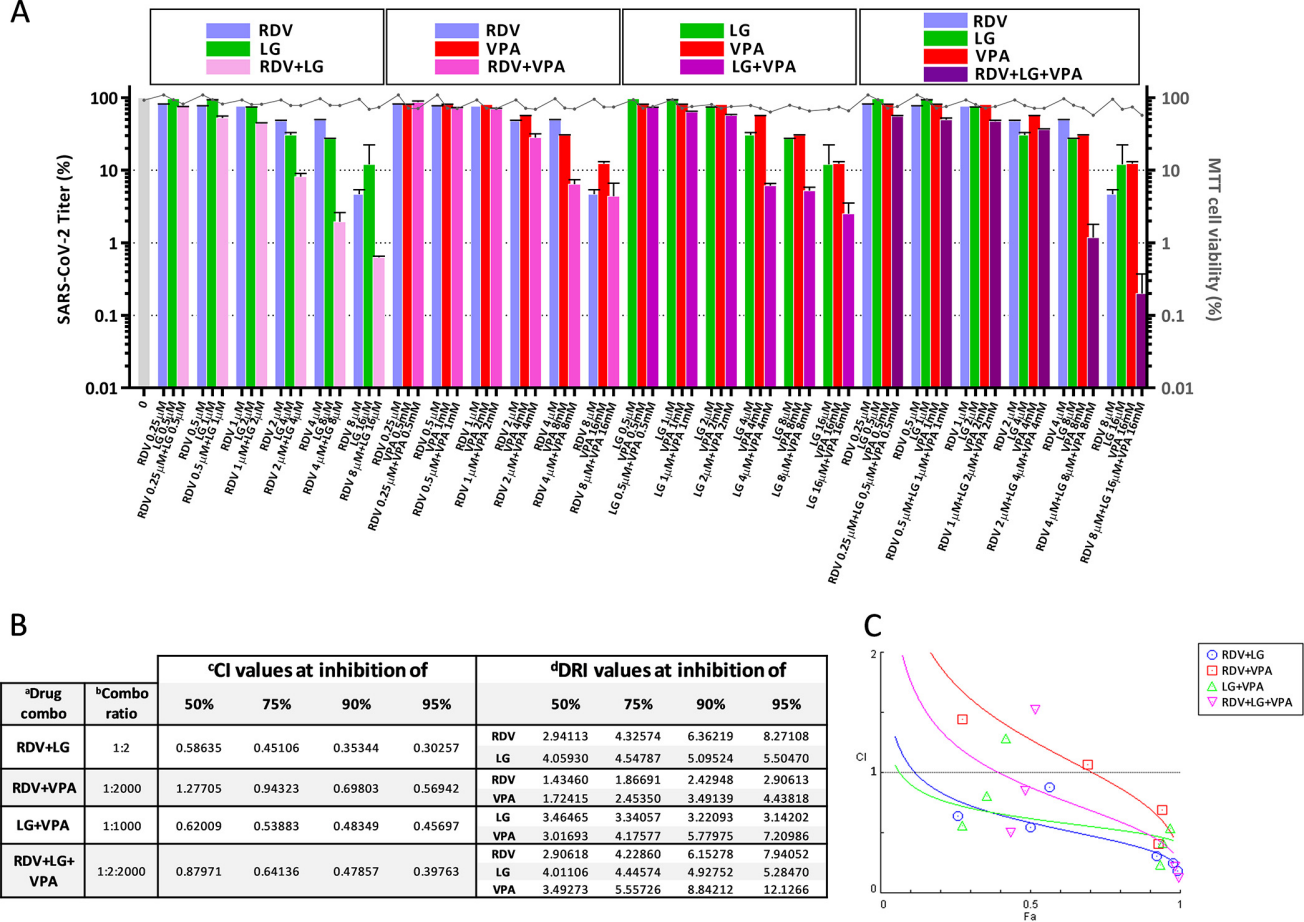
Synergistic effect of combined AVs on SARS-CoV-2 inhibition. (A) Infectious virus progeny production determined by plaque assay on triplicate cultures of Vero E6 monolayers, after infection of triplicate cultures of Calu3 2B4 cell with SARS-CoV-2 (MAD6) in the presence of one drug (LG, VPA, or RDV) or constant-ratio combinations of two or three drugs. Incubation with the AVs was carried out from 1 h before adsorption and maintained throughout the infection. (B) Synergy parameters generated by CompuSyn software (69) at 50, 75, 90, or 95% inhibition exerted by the different drug combinations (a) (drug combo), at the indicated constant-dose ratios (b) (combo ratio): synergy is determined by the combination index (CI) value (c), where CI < 1, =1, and >1 indicates synergism, additive effect, or antagonism, respectively; DRI (dose-reduction index) value (d) is folds of dose-reduction allowed for each drug, at a given degree of effect, in drug combination studies, where DRI > 1, =1, and <1 indicates favorable dose reduction, no dose-reduction, and negative dose reduction, respectively. (C) Automated inhibition effect (Fa)-CI plots generated from the median-effect equation using CompuSyn software. Fa values (0 to 1) correspond to 0 to 100% inhibition. Three replicates were made for each antiviral trial. SARS-CoV-2 titers (PFU/mL): 3.2 ± 0.2 × 10^5^ in the absence of the drugs, 6 ± 0.2 × 10^3^ at 16 μM LG, 4 ± 0.1 × 10^4^ at 16 mM VPA, 1.7 ± 0.2 × 10^3^ at 8 μM RDV, 1 ± 0.1 × 10^3^ at 16 μM LG + 16 mM VPA, 2 ± 0.1 × 10^2^ at 8 μM RDV + 16 μM LG, 1.8 ± 0.2 × 10^3^ at 8 μM RDV + 16 mM VPA and 1 ± 0.1 × 10^2^ at 16 μM LG + 16 mM VPA + 8 μM RDV.

We next studied whether a synergistic, additive, or antagonistic effect occurs when combinations of LG, VPA and RDV were used to inhibit HCoV multiplication. Thus, virus yields were determined by plaque assay, after infection of coronavirus-susceptible cell lines with the corresponding virus, in the presence of one AV (LG, VPA, or RDV) or six constant-ratio combined doses of two or the three drugs. In all cases, the range of the combined AV doses used ensured a cell viability of at least 80% (Fig. 4 to 6, right *y* axis).

First, Huh-7 cells were infected with HCoV-229E-GFP in the presence of a concentration range of 5 nM to 160 nM RDV, 0.5 μM to 16 μM LG, or 1 mM to 32 mM VPA, and six constant ratio combined doses of two or the three drugs (Fig. 4). The analysis of the virus yield at 24 hpi (Fig. 4A) indicated synergism for all drug combinations according to all parameters: CI values below 1 at effect level (Fa) ranging from 0.1 to 0.95 (10% to 95% inhibition) (Fig. 4B and C), and favorable dose reduction index (DRI) above 1 (Fig. 4B). The combination of LG and VPA against HCoV-229E-GFP (average CI of 0.23 at Fa 0.5 to 0.95) resulted highly synergistic (CI < 0.3), as observed in the combination of the three drugs (average CI of 0.28 at Fa 0.5 to 0.95). Robust synergy (0.3 < CI < 0.7) was observed in RDV + LG treatment (average CI of 0.48 at Fa 0.5 to 0.95) as well as for the combination of RDV and VPA (average CI of 0.55 at Fa 0.5 to 0.95).

We next studied the effect of drug combinations in the multiplication of the two beta coronaviruses, HCoV-OC43 and SARS-CoV-2. In the case of HCoV-OC43, the selected range of concentrations used for the infection of Huh-7 cells were 10 to 320 nM for RDV, 1 to 32 μM for LG, and 1 to 32 mM for VPA, and again, six constant ratio combined doses of two or the three compounds (Fig. 5). Virus yields at 48 hpi (Fig. 5A) revealed synergy for all drug combinations (Fig. 5B and C), but to a lesser extent than that observed against HCoV-229E-GFP. The combination of RDV and LG induced robust synergy levels (average CI of 0.66 at Fa 0.5 to 0.95) while only moderate synergism (0.7<CI < 0.85) was found for the drug combinations of LG and VPA (average CI of 0.75 at Fa 0.5 to 0.95), RDV and VPA (average CI of 0.79 at Fa 0.5 to 0.95), and for LG, RDV and VPA (average CI of 0.85 at Fa 0.5 to 0.95).

Similar results were found when testing six constant ratio concentrations of combined drugs in SARS-CoV-2-infected Calu3 2B4 cells, ranging from 0.25 to 8 μM for RDV, 0.5 to 16 μM for LG, and 0.5 to 16 mM for VPA (Fig. 6). In this case, analysis of viral titers at 24 hpi (Fig. 6A) showed that all drug combinations gave rise to robust synergy (0.3 < CI < 0.7) at Fa levels above 0.9 (average CI of 0.33 for RDV+LG, 0.51 for RDV+VPA, 0.47 for LG+VPA, and 0.44 for LG+RDV+VPA), which was also found for LG combined with RDV (average CI of 0.52) or VPA (average CI of 0.58) at Fa below 0.75 (Fig. 6B and C). Nevertheless, average CI values at Fa levels of 0.5 to 0.75 (50 to 75% inhibition) indicated a moderate synergy between the three antivirals (average CI of 0.76) against SARS-CoV-2 and an additive effect for the combination of RDV and VPA (average CI of 1.1). In any case, both the mean values of CI below 1 (average CI of 0.42 for RDV+LG, 0.87 for RDV+VPA, 0.52 for LG+VPA, and 0.59 for RDV+LG+VPA at Fa = 0.5 to 0.95) and those of DRI above 1 (average DRI of 4.31 for RDV, average DRI of 4.25 for LG, average DRI of 5.19 for VPA, at Fa = 0.5 to 0.95) showed a clear synergistic trend for all combinations of AVs against SARS-CoV-2 (Fig. 6B).

Thus, synergism has been found in the antiviral effect of the combination of LG with VPA or RDV and between the three of them, and to a lesser extent, between RDV and VPA, when acting on the three HCoVs tested. These results support the use of combination of these AVs as a strategy to inhibit coronavirus infection.

## DISCUSSION

SARS-CoV-2 first emerged in 2019 rapidly producing a global and devastating pandemic. Several vaccines have been approved and widely administered resulting instrumental in lifesaving. Nevertheless, virus variants have rapidly emerged, i.e., delta and omicron, for which current vaccination elicits less effective immune responses, making vaccine updating and improvement highly relevant for disease control. Additional important issues for COVID-19 vaccine efficacy are the duration of the protective response afforded as well as its insufficient global administration. At this stage, finding of AVs that can be used as a first line of defense to reduce morbidity and mortality is an urgent need (40). The conventional methodology for AV selection involves development of DAA drugs, an approach that has been successful against HSV, HIV-1, and hepatitis C virus (HCV) (1, 41, 42). Currently approved AV drugs that work by disrupting viral cycle have been repurposed against SARS-CoV-2 (43, 44). RDV, an adenosine analog which inhibits RNA dependent RNA polymerase (RdRp) of filoviruses, has been approved to treat SARS-CoV-2 in Europe and USA (37, 45, 46). Nevertheless, there are gaps in knowledge regarding the efficacy of RDV, such as the optimal patient population, the duration of therapy, and the clinical importance of RDV treatment effects, as well as its cost and production problems (47). In addition, two oral antiviral drugs, nirmatrelvir-ritonavir (paxlovid) and molnupiravir, have been recently authorized by the FDA for emergency use for the early treatment of mild to moderate COVID-19. Molnupiravir is a nucleoside analogue, whereas nirmatrelvir is a SARS-CoV-2 main protease inhibitor, and ritonavir is a human immunodeficiency virus type 1 protease inhibitor (48, 49). While current orally available drugs for SARS-CoV-2 are highly effective at reducing hospitalization, they are not effective for all indications, as postexposure prophylaxis. Paxlovid-resistant mutants have been found in therapy naive patients and resistance to remdesivir has also been observed *in vitro* and clinically (50). Thus, despite these repurposing efforts, no robust DAA treatment has been yet identified. An alternative antiviral approach is to target host cell pathways that are essential for virus replication. Aprotinin, a serine protein inhibitor that prevents membrane fusion, or plitidepsin, zotatifin, and rapamycin which are drugs that inhibit or modulate host protein synthesis, may have promise in treating patients with mild or moderate COVID-19 (30, 51–53). Advantages of host-targeted AVs include their potential to exert broader protection spectra against diverse viruses and viral strains. In addition, selection of virus-escape mutants that can overcome the activity of host-targeted inhibitors has been reported but the barrier to resistance is generally higher than for DAA compounds (5, 6). Similarly, that barrier to resistance can be increased by the simultaneous administration of two inhibitors directed to independent viral targets (8, 54).

We previously described the efficient inhibition of virus multiplication in several cell lines in the presence of a host-targeted AV, the antioxidant food additive LG (19, 20). We also demonstrated that VPA, another drug affecting cellular functions, used for treatment of neurological disorders, caused a drastic reduction in replication of all enveloped viruses tested (24, 25). These results led us to test their effect on human coronaviruses (HCoVs). The inhibition exerted by nontoxic concentrations of LG or VPA on HCoV multiplication resulted in a 2- to 4-log-decrease in virus yields, with IC_50_ values that ranged from a 1 to 2 μM for LG and from 5 to 9 mM for VPA. LG lead to high SI values, above 30, for all tested coronaviruses. In fact, LG inhibition of both strains (Wuhan-Hu-1 and omicron) of SARS-CoV-2 was very robust, with SI values above 50, thus LG would be able to block viral infection at doses well below its cytotoxic concentration. In the case of VPA, SI values above 7 for HCoV-229E and HCoV-OC43 indicated a moderate antiviral effectiveness, which was strong for SARS-CoV-2, regardless of the strain tested (Wuhan-Hu-1 and omicron), as indicated by SI values above 20. The similar patterns of inhibition observed for swine coronavirus TGEV and the different HCoVs, including SARS-CoV-2, supports the pan-coronavirus antiviral activity of LG and VPA, that could result in a promising strategy against emerging or reemerging coronavirus epidemics. The evidence with other virus, as well as the lack of virotoxicity of LG and VPA reported here, support that the effects of LG and VPA on cell metabolism are responsible for the antiviral effects observed (25). Nevertheless, a direct interaction of these compounds with viral components cannot be excluded. Indeed, is has been recently reported that a lead compound (3-oxo-valproatecoenzyme A conjugate) can stabilize the SARS-CoV-2 spike trimer with RBDs in the down conformation, likely impairing binding to the ACE2 receptor of the host cell (55).

The study of the effect of time of addition of the AV is relevant to understand its mechanism/s of action, as well as for designing administration patterns. In the case of VPA, the inhibitions observed were similar regardless of whether VPA was added before or after infection (25). VPA has multiple mechanisms of action, mainly affecting cellular membrane composition, that may result in impairment of virus particle production (22). Our results indicate that VPA mainly affects postentry steps in the coronavirus cycle. On the other hand, when LG was added at the time or after infection, the IC_50_ slightly increased while maintaining a consistent inhibition, indicating that the main steps inhibited by the drug were late in infection, as reported for other enveloped and nonenveloped viruses (19, 20); it also suggests that LG might affect coronavirus entry steps. We have previously shown that LG exerted an *in vivo* antiviral effect, reducing FMDV-induced mortality and viral load in mice, when administered 24 h prior to and along infection (19). The pharmacokinetic profile of LG has been studied in humans and, given the effective concentration range experimentally determined, the availability and cost of the drug is not expected to be a problem (19). VPA doses usually administered to humans are dependent on the body mass, with a therapeutic range for epilepsy treatment of 50 to 100 mg/L (about 0.3 to 0.6 mM) in plasma (25). Although the extrapolation to cellular systems seems to be complicated, these concentrations are not far from the IC_50_ here determined in cultured cells (about 5 mM), opening the possibility of potential antiviral effects *in vivo*, as proposed from clinical observations (32), particularly in the context of combined therapies such as those described here. In the case of LG, as it has been widely used as a food additive antioxidant for over 50 years, data on its metabolism, toxicological effects, and pharmacokinetics are already available. Long-term toxicity studies in rats and mice have shown that no effects were observed at doses as high as 1,000 mg kg-1 of feed (56). Likewise, we previously reported the antiviral effect of LG against FMDV in a mouse model at doses (100 mg kg-1 of body weight/day) (19) which were not found to be toxic to the animals. In any case, future studies are needed to confirm the *ex vivo* toxicity and the antiviral effect in susceptible primary cells (57) as a previous step to address the *in vivo* effect of LG and/or VPA against coronavirus infection as well as the appropriate dose and delivery route.

When specificity of LG inhibition was studied, the efficiency of LG was found to be much higher than that of the other G derivatives. Several studies have revealed that other G derivatives, such as epigallocathecin-3-gallate, prodelphinidin B-2 3′-*O*-gallate, or alkyl esters of G (mainly OG), among others, have antiviral activities against a variety of viruses (17, 18, 58–60). In the case of G alkyl esters, the antiviral activity against HSV-1 was enhanced by increasing the number of carbon in the alkyl moieties of the compounds, reaching maximum at a carbon number of 12 (LG). When tested against HCoV-229E or HCoV-OC43, OG was the only G derivative that inhibited both viruses but to a much lesser extent than LG. The inhibition exerted by LG was higher than that of G and its related compound ECG, for which an antiviral activity higher than that of LG had been proposed based on molecular docking studies against five selected nonstructural proteins of SARS-COV-2 (33), supporting the need of cellular assays to confirm *in silico* studies (10).

The inhibition exerted by combinations of AV compounds may be stronger than either drug alone, a phenomenon known as synergy that can enhance the efficacy of antiviral therapies. It has been proposed that combinations of AV drugs are more likely to be synergistic if they include different types of compounds, have independent mechanisms of action and/or affect different stages of virus life cycle (10). AV synergy has been previously shown for Ebola and other viruses such as SARS-CoV-2 (61). Combining molnupiravir and paxlovid conferred synergistic suppression of SARS-CoV-2 infection in human lung cells and in a mouse model (62). Combination regimens that include DAAs and host-targeted antivirals can enhance antiviral potency, reduce the emergence of variants, and lower the dose of each component in the combination. Recent studies show the synergistic potential of combining host-targeting antivirals that either target SARS-CoV-2 entry (camostat, nafamostat, and avoralstat) or replication (brequinar) with the DAA molnupiravir, to block SARS-CoV-2 infection with the DAA molnupiravir, to block SARS-CoV-2 infection in Calu-3 lung epithelial cells (63). Although LG and VPA target cellular metabolism, their known mechanisms of action are different and could affect multiple steps of the viral cycle. LG is known to protect cells from oxidative stress by inhibiting enzymes involved in lipid peroxidation and increasing expression of antioxidant genes and due to its hydrophobic properties it can disrupt biomembranes and cause protein inactivation (64), while VPA has been shown to alter a variety of signaling pathways, including an increase in GABA neurotransmission, inhibition of histone deacetylases (HDACs), inhibition of glycogen synthase kinase beta (GSK3β), or attenuation of phospholipid signaling (22). Thus, we hypothesized that LG and VPA could be good candidates for drug combination studies. Indeed, a notable synergism for combinations of LG and VPA against HCoV-229E, SARS-CoV-2, and HCoV-OC43 according to Chou-Talalay CI and DRI values (CI < 1, DRI > 1), was observed. LG synergized with VPA at low concentrations (up to 4 μM for LG and 8 mM for VPA) when tested against HCoV-229E or HCoV-OC43, while the most robust synergism against SARS-CoV-2 was observed at the highest concentrations of both drugs tested (4 to 16 μM for LG and 4 to 16 mM for VPA). This result is relevant to determine the appropriate dosing combination for *in vivo* studies.

Several synergistic combinations have been reported between RDV and other AVs, such as nitazoxanide, although striking antagonism was observed *in vitro* in the combination of RDV and hydroxychloroquine, the other drug approved by the US Food and Drug Administration (FDA) emergency use authorization (EUA) to treat COVID-19 (10, 43). Interestingly, a notable synergism against the three HCoVs studied was observed when combining RDV with LG and/or VPA. In the case of HCoV-229E and HCoV-OC43, the strongest synergism was found when the three drugs were combined, though RDV+LG and RDV+VPA combinations also demonstrated robust synergism. The window of concentrations with greater synergy corresponded in all cases to low concentrations of the three drugs, below 80 nM for RDV, 8 μM for LG, and/or 8 mM for VPA. For SARS-CoV-2, a robust synergy was found between RDV and LG, while the combination of RDV and VPA turned out to be additive at low concentrations of the two drugs but synergistic at high concentrations. The strongest synergy was observed in the combination of the three drugs and in all cases and unlike in the other HCoVs, the synergy was greater at the medium and high concentrations assayed (RDV, 2 to 8 μM; LG, 4 to 16 μM; VPA, 8 to 16 mM).

Our results revealed strong synergy in the inhibition of the multiplication of three HCoVs, including SARS-CoV-2, between LG and VPA and to a lesser extent in their combination with RDV. It is important to note that some of the drug combinations found are dose-dependent in cultured cells and determining an appropriate clinical dosing combination is more difficult *in vivo* and requires experiments with animal models to determine toxicity thresholds and the protection they can confer against virus challenge. Nevertheless, the finding of synergy in different human cell lines and against three HCoVs support the testing of these drug combinations in animal models. Being aware of the potential limitations introduced by immortalized cell lines, most of our work has been conducted using Calu3 2B4 cells, whose origin and phenotype is close to the respiratory target cells of SARS-CoV-2 (65). These cells are physiologically relevant as derive from human lung epithelium and are known to minimize deletion of the furin-cleaveage site at the spike protein, which could lead to inconclusive results (66).

Our findings reinforce the interest of testing the effect of host-targeted compounds such as LG and VPA, as a first line of disease defense or as a vaccine complement to minimize the gap in antibody-mediated protection evoked by vaccines, either in the case of SARS-CoV-2 and its emerging variants, or for other possible new viruses. The possibility of combined treatments, including LG, VPA, and the DAA compound RDV might result in interesting possibilities for developing COVID-19 therapies.

## MATERIALS AND METHODS

### Cells and viruses.

Vero E6 (VERO C1008 [Vero 76, clone E6, Vero E6], African green monkey kidney epithelial cells, kindly provided by Luis Enjuanes at CNB, Spain), Huh-7 (male hepatoma tissue cells, kindly provided by Esteban Domingo at CBMSO, Spain) (67), ST (swine testis cells, ATCC, CRL-1746), WSL (wild swine macrophage cell line, kindly provided by Günther Keil at Friedrich-Loeffler Institut, Germany), and Calu3 2B4 clone (human bronchial ephitelial cells with increased ACE-2 expression, kindly provided by Kent Tseng at UTMB, USA and Luis Enjuanes at CNB, Spain) (65) cell lines were grown at 37°C in DMEM medium supplemented with 5%, 10%, or 20% fetal calf serum, while HCT-8 ([HRT-18] ATCC CCL-244, human colorectal adenocarcinoma) cells were grown at 37°C in ATCC-formulated RPMI-1640 medium supplemented with 10% horse serum. TGEV (kindly provided by Luis Enjuanes at CNB, Spain) stock was produced and titrated in ST cells. HCoV-229E-GFP (kindly provided by Volker Thiel at University of Bern, Switzerland, and Luis Enjuanes at CNB, Spain) was propagated and titrated in Huh-7 cells while HCoV-OC43 virus (ATCC VR-1558) production and titration was performed in HCT-8 cells, as previously described (68). SARS-CoV-2 isolate MAD6 (Wuhan-Hu-1-like strain, kindly provided by Luis Enjuanes at CNB, Spain) was propagated in Calu3 2B4 cells while its titration was done in Vero E6 cells; SARS-CoV-2 isolate 39896362_AG (omicron BA.1-like strain, kindly provided by Ana Grande at University of Málaga, Spain) was propagated and titrated in Vero E6 cells. To measure SARS-CoV-2 titers by plaque assay, viruses were subjected to serial 10-fold dilutions, added to confluent Vero E6 cell monolayers, and incubated for 60 min at 37°C. Medium was removed and cells were overlaid with DMEM containing 0.3% carboxymethyl cellulose (CMC) and 2% FBS. At 72 hpi., cells were fixed with 10% formaldehyde and stained with crystal violet.

### AVs and cell viability assays.

LG, G, propyl gallate (PG), and octyl gallate (OG) (Sigma-Aldrich) were solubilized in 100% ethanol, epicatechin gallate (ECG, Sigma-Aldrich) in 15% ethanol, valproic acid (VPA, Sigma-Aldrich) in DMEM, and RDV (GS-5734, MedChemExpress) in 100% DMSO. AV solutions were diluted in DMEM to prepare working stocks. Cell viability determinations were performed by the MTT (3-[4,5-dimethylthiazol-2-yl]-2,5 diphenyl tetrazolium bromide) assay, as previously described (20). Briefly, cell monolayers were grown on 96-well plates before the addition of the AV or AV combination (six wells for each dose). After 24 to 48 h of incubation, MTT-containing culture medium was added, and cells were incubated for 2 h and treated with SDS-containing lysis solution. Absorbance at 550 nm was determined after 15 min of incubation, and average values (from 6 wells) obtained were subtracted from the background levels (in the absence of cells) and compared with the data scored in the absence of AV or AV combination (100% viability). Incubation temperature was 33°C for HCoV-229E-GFP and HCoV-OC43-susceptible cell lines and 37°C for SARS-CoV-2 susceptible cell lines. CC_50_ values (concentration of AV yielding a 50% of cell viability) shown in Table 1 were determined from the viability curves of each cell line with each AV or AV combination (right *y* axis of Figs. 1 to 6).

### Direct virucidal effect.

To determine a possible direct virucidal effect of the AVs on virus particles, suspensions of different viruses (10^5^ PFU) in 0.9 mL of culture medium were incubated with 0.1 mL of LG (0 to 300 μM) or VPA (0 to 300 mM); controls with viruses incubated in 2% ethanol in the absence of the drug were included. After 1 h of incubation at room temperature, samples were diluted (1:1,000) and titrated by plaque assay on virus-susceptible cell monolayers (Huh-7 for HCoV-229E-GFP, HCT-8 for HCoV-OC43, and Vero E6 for SARS-CoV-2 samples). Virus titers in AV-treated duplicate samples were compared with those obtained in virus samples incubated in the absence of the drug.

### HCoV infections and antiviral treatment.

Huh-7 (for HCoV-229E-GFP and HCoV-OC43), Calu3 2B4 (for SARS-CoV-2 Wuhan-Hu-1 strain), or Vero E6 (for SARS-CoV-2 omicron strain) cell monolayers were grown on multiwell plates in triplicate cultures, preincubated for 1 h with different AVs at 37°C, and then infected with the corresponding virus, at an MOI of 2 PFU/cell, in a reduced volume of adsorption medium containing the AV, for 1 h (for SARS-CoV-2) or 2 h (for HCoV-229E-GFP and HCoV-OC43). The virus inoculum was removed, and cells were washed twice with medium before the addition of drug-containing fresh medium (supplemented with 2% FCS). Cultures were then incubated at 33°C for 24 h for HCoV-229E-GFP or for 48 h for HCoV-OC43, or at 37°C for 24 h for SARS-CoV-2 assays. Total virus (intracellular and extracellular) production was evaluated by plaque assay on the corresponding virus-sensitive cells (Huh-7 for HCoV-229E-GFP, HCT-8 for HCoV-OC43, or Vero E6 for both SARS-CoV-2 strains). From the inhibitor-response curves of each AV on each virus yield, generated using three-parameter fits 50% inhibitory concentration (IC_50_) and selectivity index (SI=CC_50_/IC_50_) were determined.

### Time of addition assay.

To address the possible step of the virus cycle targeted by each AV, susceptible cell monolayers in triplicate cultures were infected with virus at an MOI of 2 PFU/cell. After adsorption (1 h for SARS-CoV-2 Wuhan-Hu-1 strain or 2 h for HCoV-229E-GFP and HCoV-OC43), the inoculum was removed, and cells were further incubated. At different hours postinfection (hpi), that is 1 h before adsorption (−3 hpi for HCoV-229E-GFP and HCoV-OC43, or −2 hpi for SARS-CoV-2), at the time of infection (0 hpi) and 2 h after infection (2 hpi), AVs were added to triplicate wells at increasing concentrations, and the cultures were further incubated until 24 hpi (for HCoV-229E-GFP and SARS-CoV-2) or 48 hpi (for HCoV-OC43), collected, and total virus titers determined by plaque assay in the corresponding coronavirus-susceptible cell lines.

### Synergy studies and statistical analysis.

Data handling and analysis were performed using Graph Prism 6.01 software; IC_50_ values were determined by inhibitor-response curves generated using three-parameter fits. Synergism between two or three of the combined drugs (LG, VPA, and RDV) was analyzed using CompuSyn software (ComboSyn, Paramus, NJ, 2005) (69), based on the multiple drug effect analysis of Chou and Talalay (7, 69–71), which provides the theoretical basis for the combination index (CI)-isobologram equation that allows quantitative determination of drug interactions, where CI < 1, =1, and >1 indicates synergism, additive effect, and antagonism, respectively. DRI (drug combination index) is defined as folds of dose-reduction allowed for each drug, at a given degree of effect, compared to the effect of the drug alone, where DRI > 1, =1, and <1 indicates favorable dose reduction, no dose-reduction, and negative dose-reduction, respectively (70).
