# Supplementary material for: Inhibition of Human Coronaviruses by Combinations of Host-Targeted and Direct-Acting Antivirals
Source: Antimicrob Agents Chemother. 2023 Mar 28;67(4):e01703-22. doi: 10.1128/aac.01703-22 (PMC10112268; doi:10.1128/aac.01703-22)
Supplement: Supplemental file 1 — Supplemental material. Download aac.01703-22-s0001.pdf, PDF file, 0.6 MB [file aac.01703-22-s0001.pdf]

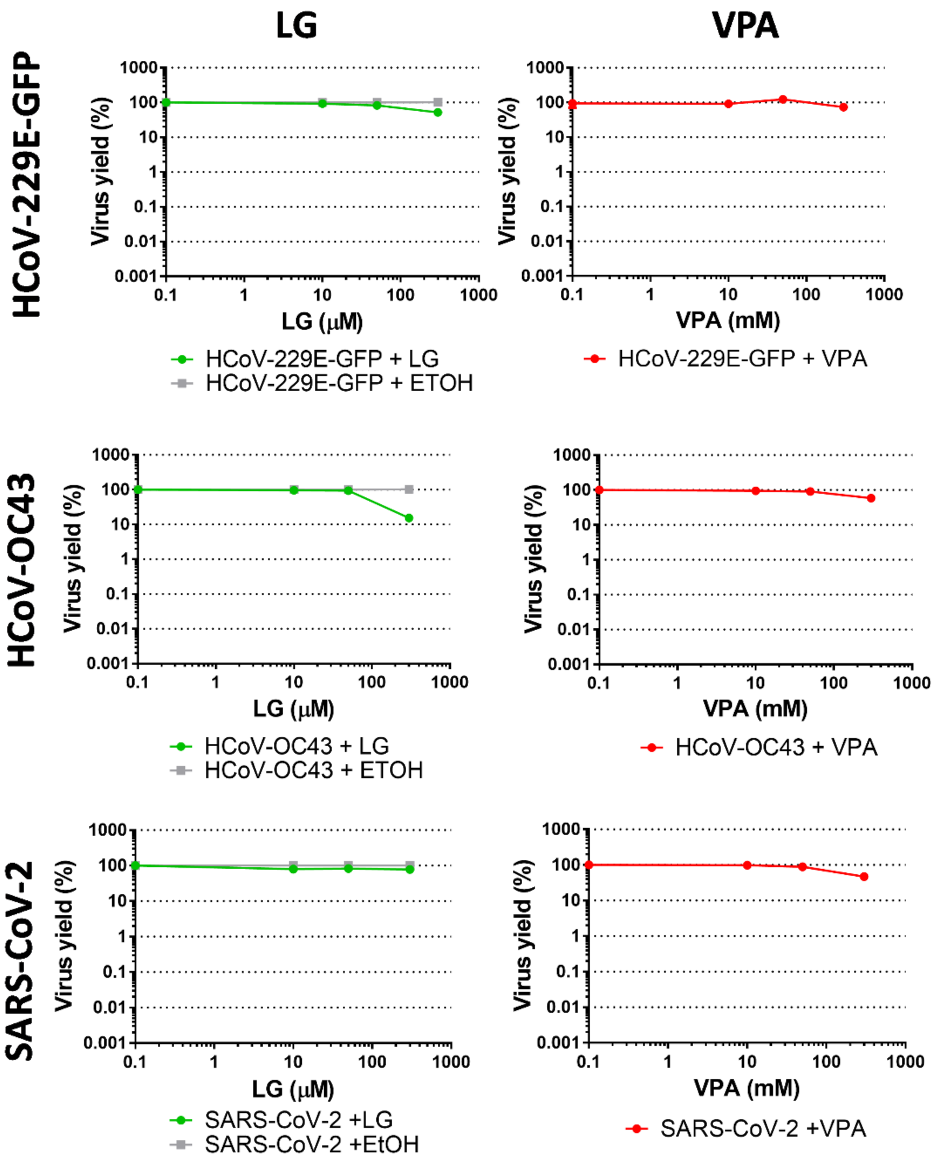

Figure S1. LG and VPA virotoxicity on HCoV-229E-GFP, HCoV-OC43 and SARS-CoV-2. Triplicate virus suspensions were incubated for 1 h at room temperature with LG (0-300 μM), VPA (0-300 mM); controls with viruses incubated in 2% ethanol in the absence of LG were included (solvent). Samples were then diluted (1:1000) and titrated by plaque assay on triplicate cultures of Huh7 cells for HCoV-229E-GFP, HCT-8 cells for HCoV-OC43 or Vero E6 cells for SARS-CoV-2. [HCoV-229E-GFP titers (PFU/ml): 3.7±0.2 x 10^5 in the absence of the drugs, 2±0.2 x 10^5 at 300 μM LG or 3±0.2 x 10^5 at 300 mM VPA. HCoV-OC43 titers (PFU/ml): 3.8±0.2 x 10^5 in the absence of the drugs, 7±0.2 x 10^4 at 300 μM LG or 2±0.2 x 10^5 at 300 mM VPA. SARS-CoV-2 titers (PFU/ml): 2.1±0.2 x 10^5 in the absence of the drugs, 1.6±0.1 x 10^5 at 300 μM LG or 1±0.2 x 10^5 at 300 mM VPA].

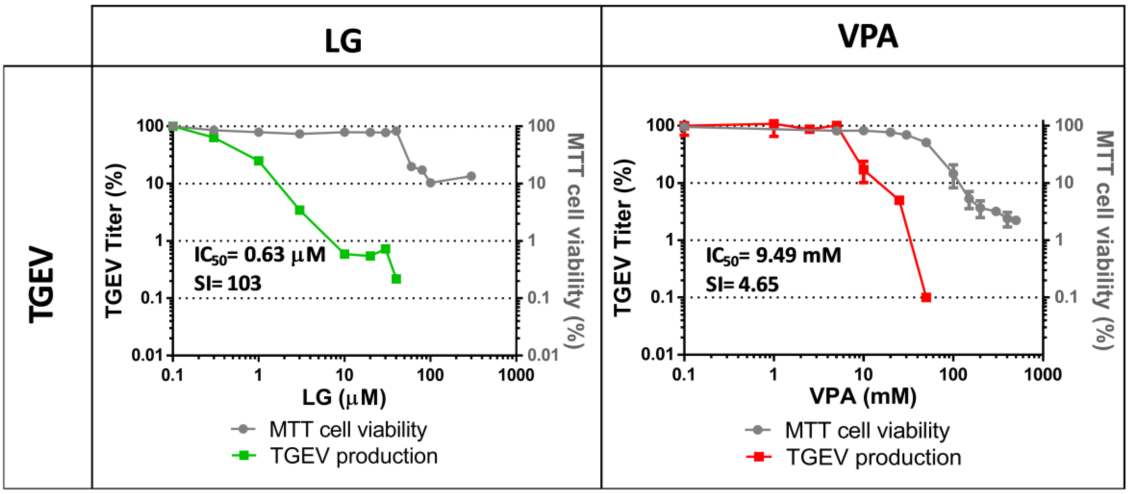

Figure S2. Antiviral effect of LG or VPA against TGEV. The inhibitory effect of LG or VPA was assayed on triplicate cultures of TGEV-infected WSL (wild swine macrophage cell line) or ST (swine testis) cells, respectively. After 1 h-pre-incubation with AVs, triplicate cultures were infected with the corresponding virus, at a MOI of 2 PFU/cell, in a reduced volume of medium containing the AV, for 2h, virus inoculum removed and cells washed twice with medium, before the addition of drug-containing fresh medium (supplemented with 2% FBS). Cultures were then incubated for 24 h at 37ºC; total virus (intracellular and extracellular) production was evaluated by plaque assay on triplicate cultures of ST cells. Left y-axis represents infectious virus progeny at 24 hpi and right y-axis, cell viability determined by MTT assay of each corresponding cell line. IC_{50} values and SI values are displayed. [TGEV titers (PFU/ml): 1.1±0.1 x 10^8 in the absence of the drugs, 2.4±0.3 x 10^5 at 40 µM LG and 1.1±0.1 x 10^5 at 50 mM VPA].
